# Development of a CHO cell line for stable production of recombinant antibodies against human MMP9

**DOI:** 10.1186/s12896-022-00738-6

**Published:** 2022-03-07

**Authors:** Jina Ryu, Eun-Jung Kim, Joo-Kyung Kim, Tai Hyun Park, Byung-Gee Kim, Hee-Jin Jeong

**Affiliations:** 1 Interdisciplinary Program in Bioengineering, Seoul National University, Seoul, 08826 Republic of Korea; 2 School of Chemical and Biological Engineering, Institute of Chemical Processes, Seoul National University, Seoul, 08826 Republic of Korea; 3 BioMAX/N-Bio Institute, Institute of Bioengineering, Seoul National University, Seoul, 08826 Republic of Korea; 4 Department of Biological and Chemical Engineering, Hongik University, Sejong, 30016 Republic of Korea

**Keywords:** Matrix metalloproteinase 9, Recombinant antibody, CHO cell line

## Abstract

**Background:**

Human matrix metalloproteinase 9 (hMMP9) is a biomarker in several diseases, including cancer, and the need for developing detectors and inhibitors of hMMP9 is increasing. As an antibody against hMMP9 can be selectively bound to hMMP9, the use of anti-MMP9 antibody presents new possibilities to address hMMP9-related diseases. In this study, we aimed to establish a stable Chinese hamster ovary (CHO) cell line for the stable production of antibodies against hMMP9.

**Results:**

Weconstructed recombinant anti-hMMP9 antibody fragment-expressing genes and transfected these to CHO cells. We chose a single clone, and successfully produced a full-sized antibody against hMMP9 with high purity, sensitivity, and reproducibility. Subsequently, we confirmed the antigen-binding efficiency of the antibody.

**Conclusions:**

We developed a novel recombinant anti-hMMP9 antibody *via* a CHO cell-based mammalian expression system, which has a high potential to be used in a broad range of medical and industrial areas.

**Supplementary Information:**

The online version contains supplementary material available at 10.1186/s12896-022-00738-6.

## Introduction

Matrix metalloproteinase 9 (MMP9) is one of the 23 family members of MMP that performs a central function in modulation of cellular homeostasis [1]. Dysregulated MMP9 expression and its abnormal activity are involved in pathological processes that contribute in chronic inflammation, tumorigenesis, and metastasis [2–4]. Because of its correlation with diseases, such as ulcerative colitis and colorectal cancer, MMP9 has been detected as a biomarker in numerous diseases including cancer and believed to be a potential target for therapeutic intervention [5]. Initial efforts to develop MMP9 inhibitors were mainly focused on the use of metal chelators [6]. Even though the methods provided highly potent inhibitors, the dose-limiting toxicity and side effects are still concerned [7, 8]. Additionally, those inhibitors were not capable of differentiating between MMP family members. Thus, new methods were necessary to identify a selective MMP9, and antibodies against MMP9 seemed to be encouraging options to the chelator inhibitors [9, 10]. As MMP9 can be blocked selectively with an anti-MMP9 antibody, the field of MMP9 interference by way of its inhibitory antibody presented new possibilities on the road to address MMP9-related diseases [10, 11]. Paemen *et al*. created an anti-MMP9 monoclonal antibody through hybridoma technology and showed its activity [9, 12]. However, a hybridoma cell-based expression possesses stability and reproducibility issues with the antibody, which impeded its use for large-scale production. Further, at least a third of clonal hybridoma cell lines might hold additional antibody genes, leading to the generation of an amalgamation of antibodies with various affinities to the target antigen. Consequently, it is essential to have immunized syngeneic animals for creating a new batch of hybridoma cells, engendering batch-to-batch fluctuation. Recombinant antibody production technology has been cultivated and implemented in the biopharmaceutical industry, as it elicits high consistency and reproducibility. Appleby et al. created a humanized monoclonal antibody against human MMP9 (hMMP9) and described its enzymatical activity by investigating the three-dimensional structure (PDB: 5th9) [10]. Even though the sequence of this antibody was disclosed as well as the recombinant antibody was opportunely generated, the yield was not adequate for a large-scale expression. Hence, additional development of the production performance was imperative.

**Fig. 1.**
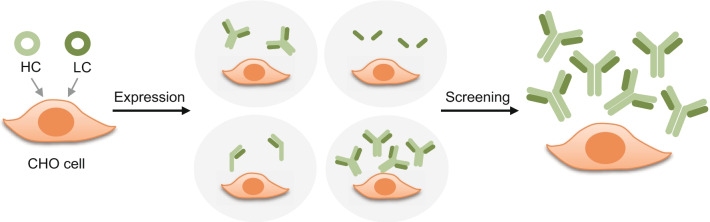
Schematic representation of the production of CHO cell-based anti-hMMP9 monoclonal antibody. HC and LC indicates heavy chain and light chain, respectively.

Mammalian cell expression systems are the preference to produce eukaryotic proteins, as they facilitate post-translation modifications, which are critical for the correct folding of proteins. Glycoproteins including antibodies are usually synthesized in mammalian cells, whereas microbial hosts, such as *E. coli*, are without the requisite machinery to synthesize proper glycosylated forms [13, 14]. Either transient transfection or development of a stable cell line is the approach to produce antibodies in mammalian cells, and those production outputs are typically much higher than those by *E. coli*-based production. It is possible to generate secreted mammalian proteins in a large-scale format in mammalian cells, such as human embryonic kidney (HEK) and Chinese hamster ovary (CHO) cells. Transient cell lines, such as HEK cells, have been utilized because of their high transfection effectiveness and antibody production capacity. It has been demonstrated, however, that there are variances in the manufacturability, affinity, and efficacy of antibodies developed in HEK cells compared with those created by stably transfected CHO cells [15]. A stable CHO cell line is extensively used in adept antibody production because it performs human-compatible post-translational modifications and does not spread human pathogenic viruses [16]. Thus, CHO cell expression has become predominant for fabricating owing to its capability to produce large quantities of monoclonal antibody or antibody fragments with a constant quality [17, 18]. Indeed, as the adeptness to execute the indispensable protein folding, assembly, and post-translational modifications such as glycosylation of CHO cell-based creation is high, there is a benefit that the antibody prepared is biochemically equivalent to those of human forms for greater product efficacy and safety. In this research, we aimed at establishing a stable CHO cell line for the generation of a recombinant anti-hMMP9 antibody, potentially important as a therapeutic product for cancer and disorders. We composed a recombinant anti-hMMP9 monoclonal antibody-expressing gene encoding the heavy chain (HC) or the light chain (LC) and transfected these to CHO cells. We screened the clones and chose a single clone. Subsequently, we purified antibodies from the expression medium and established the antigen-binding efficiency of the antibody (Fig. 1).

## Results and discussion

**Fig. 2.**
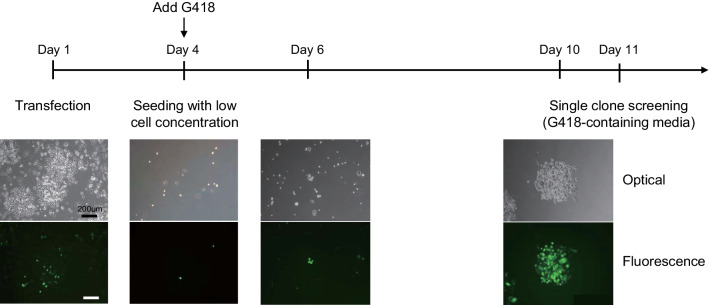
Timeline of growing anti-hMMP9 antibody-expressing CHO cell line. GFP-transfected control CHO cell line was observed by fluorescence microscopy for specifying the growth status of gene-transfected cells. Scale bar = 200 μm.

To create a recombinant anti-hMMP9 antibody-expressing genes, we performed a codon optimization of the genes encoding the HC and LC of the anti-hMMP9 antibody, for expressing the genes in CHO cells, and separately introduced the genes to mammalian expression vectors (Table 1). Herein, we used a GS-5745 gene [10] as a template for amplifying the variable region of heavy chain (VH) and variable region of light chain (VL) of an anti-hMMP9 antibody because it is recognized that the expressed GS-5745 antibody is selectively bound to the pro-catalytic domains of hMMP9 with a KD of 0.168 ± 0.117 nM and IC50 of 0.218 ± 0.040 nM [10]. For the purpose of developing a genetic construct for expressing a full-sized antibody in a CHO cell, we utilized a pcDNA3.1(−) vector, which is a broadly used mammalian expression vector. As CH and CL genes, a human IgG4 constant region and a human kappa constant region, respectively, were employed. Subsequent to the co-transfection of both HC and LC expression plasmids into CHO cells, we tandemly screened the transfected cells. We observed the cells through a microscopy a day after transfection and affirmed that the cells were well amplified. To grow the cell line, we supplemented an antibiotic geneticin (G-418) to the media since pcDNA3.1(−) includes a neomycin resistance gene (*neo*). In the presence of 600 μg/mL G-418, cells were selected and expanded. We observed the cells by utilizing a microscopy three days after, and the transmission image displayed the adequacy of the antibiotic selection. At that point, we inserted no reporter gene with the vectors, did not label the IgG using GFP, and expressed IgG chains separately to impede a low yield of antibody production, as well as to decrease experimental steps for truncating the expressed reporter molecule from the antibody. Therefore, it was difficult to promptly confirm the transfection efficiency and to selectively differentiate the antibody gene-transfected clones. Hence, we transfected a GFP-expressing gene to additional cells and used the GFP-expressing transfectants as a control for confirming the transfection efficiency and optimizing the G-418 concentration forantibody-expressing gene-transfected cells (Fig. 2). Namely, the GFP plasmid was designed using a pcDNA 3.1(−) as a backbone, which was the same as the backbone used for generating a target hMMP9 antibody. The use of these two plasmids was likely to have similar expression rates because promoter, combination of RNA polymerase binding site, and response elements were identical. Therefore, although GFP was expressed separately, it was considered that the expression of the GFP gene could be used as a sufficient material to compare its expression rate and appropriate antibiotics concentration to the one of antibody expression gene. When we observed the cells by employing a fluorescence microscopy, the fluorescence image of the cells showed numerous fluorescent spots but not all cells. Accordingly, we seeded a low concentration of cells for three days post transfection and added G-418. As a result, we consistently cultured the cells for an additional seven days and verified that several colonies were generated. Following that, the clones were isolated by limiting dilution cloning [6]. Namely, we selected 24 single clones with an analogous shape and size to the fluorescence cells in G-418-containing media from the non-GFP-transfected cells, thus signifying that the selected clones have high potential to be antibody-expressing gene-transfected cells. Consequently, we seeded the selected clones to each well of a 24-well plate. We then cultured the cells for seven days and selected six leading clones (#3, #6, #10, #13, #14, and #18) from the 24 clones that revealed high growth circumstances. After this initial screening, we transferred each clone to flasks and cultured the cells for a further seven days.

**Table 1.** The sequences of H chain and L chain of recombinant anti-hMMP9 antibody

Domain	Nucleotide sequence
VH	caagtccaattgcaagaatcaggaccaggactcgtcaaaccttctgaaacactctctctcacgtgtacggtctctggattttcacttttgtcttatggggtacattgggtacggcaaccacctggaaaagggttggaatggctcggtgttatatggactggcggaacaacaaactataactcagctctcatgagtcggtttacaatttccaaggatgattctaagaatactgtttatctcaaaatgaatagtctcaagacagaggatacggcaatttactactgtgcacgctattattatggaatggactactggggacaaggaactctcgtcacagtctcttca
CH	gcttctacaaaagggcctagtgtctttccgcttgctccatgttcacggtcaacatctgaaagtacggcagctttgggatgtctcgtaaaagattattttcctgaacctgtcacagtctcctggaatagtggggcgctcacatccggagtacatacgtttcctgcagttcttcaatcttctgggttgtattctttgtcatcagttgttactgtcccatcatcctctcttggtaccaaaacatatacatgtaatgtcgatcataaaccttccaatactaaagtagacaaacgggttgaattcaaatatggaccaccttgtccctcatgcccagctccggaatttctcggcgggccgtcagtatttctctttccccctaaacctaaagatacacttatgatatcacggactccagaagtcacatgtgttgtagttgatgtctctcaagaagatccagaagtgcaatttaattggtatgttgatggggtagaagttcataatgccaaaacaaaacctcgcgaagaacaatttaattccacttatcgggtcgttagtgtcctcacagtactccatcaagattggcttaacgggaaagaatacaaatgtaaagtttctaacaaaggtttgccttcatcaatagagaagactatttccaaagcaaaggggcaacctcgagaacctcaagtttacacattgccaccatcccaagaagaaatgacaaagaatcaagtttctcttacttgtctcgtcaaaggattttatccgtccgatatagcagttgaatgggaaagtaatgggcaaccggaaaataattacaaaactacaccgcctgttttggattcagatggaagtttcttcctctattcaagattgactgtagataaatccagatggcaagaaggtaatgtcttttcatgttccgtcatgcatgaagcgcttcataatcattatactcaaaagtctctttcattgtccctcggtaaa
VL	gatatccaaatgacccagagtccttcatcactctcagccagcgtcggtgacagggtaactattacttgcaaggcctcacaggacgtccgaaacacagttgcctggtaccagcagaaacccgggaaggcgcccaagctgctgatctacagcagctcatacaggaacaccggtgtgcccgacagattcagtggcagcggcagtggcaccgacttcaccctgaccatcagctctcttcaggccgaggacgtggctgtctactactgccagcagcactacataacaccatacacgttcggtggcggaacgaaggttgagatcaagaggactgttgca
CL	gccccttctgtgttcattttcccaccgtctgacgagcagctcaagtcaggcaccgcgagcgtggtgtgcttgctgaacaacttttaccctcgcgaggccaaggtgcagtggaaggtggacaacgccctgcagtccggtaacagccaggagagtgttaccgagcaggactctaaggactcaacgtacagcctcagttctactctcaccctgtcaaaggctgactacgaaaagcacaaggtatacgcctgcgaggttacccaccagggtctgagctctcctgtgaccaagagcttcaaccggggcgagtgc

As the CHO cells secreted proteins to the extracellular medium, we collected each medium after cultivation and purified the antibodies from the supernatants by means of PA bead purification. After concentrating each purified antibody by employing an ultrafiltration system, we addressed the concentration of antibodies with a nanodrop and determined the samples by SDS-PAGE. As we expected, the concentration of each antibody was not insignificant but low at this beginning stage (1.17, 0.78, 3.30, 1.31, 1.98, and 2.28 μg/mL for #3, #6, #10, #13, #14, and #18, respectively), denoting the necessity of subculture for enhancing the concentration of antibodies. SDS-PAGE analysis of the reduced condition leads to the bands corresponding to HC and LC with the expected sizes of 55 and 28 kDa, respectively. Not only that, the band corresponding to the full-sized antibody with the expected size of 166 kDa was seen on the gel in the non-reduced condition. By comparing the bands of reduced and non-reduced samples, it was substantiated that the antibodies were expressed with correct folding (Additional file 1: Fig. S2). Notwithstanding, on the gel of the non-reducing condition, we found not only a full-sized antibody but also the band around 25 kDa, proposing the possibility of the presence of solely LC fragments in the sample. Considering that the concentration of the sample was low, we could not absolutely identify from the gel whether the band around 25 kDa indicated an excess LC fragment or not. Although Western blotting (WB) could be used for identifying the fragment owing to the sensitivity of WB being higher than that of CBB-staining, this matter was explained eventually by conducting SDS-PAGE analysis utilizing a sample with increased concentration, as indicated below.

**Fig. 3.**
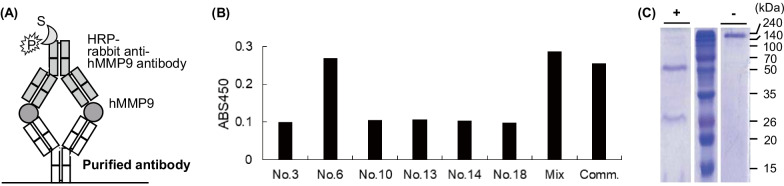
**A** Schematic representation of the sandwich ELISA for selecting the positive clones of antibodies. **B** ELISA signal with various colonies of antibodies. Mix and Comm. indicates a mixed antibody prior to selection and a commercial anti-hMMP9 antibody, respectively. **C** SDS-PAGE analysis of #6 antibody after (+) or before (–) protein reducing through 100 mM DTT and heating. We displayed a cropped gel in Fig. 3C. The original gel was presented in Additional file 1: Fig. S6.

We executed a sandwich ELISA to verify the antigen-binding activity of antibody purified from six selected CHO clones. We seeded each antibody to a 96-well plate and added a commercial full-sized recombinant hMMP9 as an antigen. Next, we added a commercial HRP-conjugated anti-hMMP9 antibody as a detection antibody, followed by the addition of a substrate, and measured the absorbance intensity of the product (Fig, 3A). At that time, we employed both a purified ‘mixed’ antibody, which was acquired from the cell supernatant before the screening, and a commercial anti-hMMP9 antibody as positive controls. The antigen-binding efficiency of each antibody differed because the characteristics of each antibody from mammalian cell expressions, such as post-translational modification including glycosylation, folding, and assembly might vary. We verified positive responses from all antibodies. Among these, clone #6 showed the highest titer, which was approximately the same as the mixed sample, as well as the commercial antibody (Fig. 3B). On the basis of the result, we chose clone #6 for the continued analysis and sub-cultured the clone in a shaking flask to secure larger amounts of the antibody. Afterward, we collected the medium and purified antibody from the supernatant. When we loaded the purified antibody to an SDS-PAGE gel, clear bands were observed from both reducing (28 kDa and 55 kDa for LC and HC, respectively) and non-reducing conditions (166 kDa for full-sized antibody), substantiating the finding that the antibody was effectively expressed with correct folding and the excess proteins, such as LC fragments and half antibody, which could be contained in the culture medium were eliminated after the purification (Fig. 3C).

**Fig. 4.**
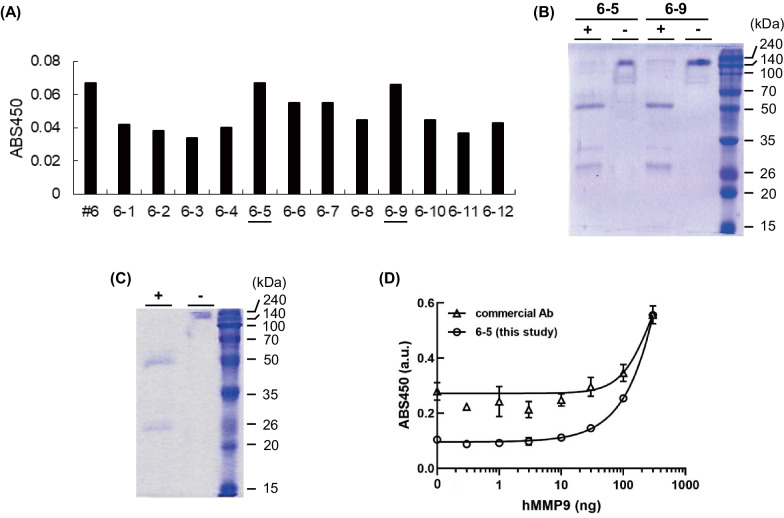
**A** ELISA signals of anti-hMMP9 antibody from various colonies following a second round of selection; **B** SDS-PAGE analysis of expressed anti-hMMP antibodies from #6–5 and #6–9 clones with (+) or without (−) protein reducing; **C** SDS-PAGE analysis of #6–5 clone after PA affinity chromatography with (+) or without (−) protein reducing; and **D** ELISA signals of #6–5 clone and commercial anti-hMMP9 antibody with different concentrations of hMMP9. Error bars represent ±1 standard deviation (SD) (n = 3). We displayed cropped gels in Fig. 4B, C. The original gels were presented in Additional file 1: Fig. S6.

As a second round of selection, we additionally picked up 12 single clones from the sub-cultured colony #6 and then singly seeded them to a 24-well plate. Following 1 week cell cultivation, we gathered the medium and purified antibody from the supernatant. Thereafter, we conducted a sandwich ELISA and confirmed that the fifth and ninth colonies (#6–5 and #6–9) showed the highest responses (Fig. 4A). For this reason, we further cultured the two colonies separately in a flask with 40 mL medium and then purified the antibody utilizing PA beads followed by size-exclusion chromatography (Additional file 1: Fig. S3, S4). We validated that antibodies were successfully expressed from both #6–5 and #6–9 clones (Fig. 4B) from the SDS-PAGE result at reducing and non-reducing conditions. The yield of the purified antibodies from #6–5 and #6–9 clones was calculated as 1.58 and 1.78 mg from 1 L culture, respectively. We used ProCHO5 as a culture medium by considering the production cost. However, the use of other CHO cell culture media, such as ActiCHOP, BalanCD CHO [19], can be attempted to improve the yields. Furthermore, more detailed optimization of the experimental conditions for cell culture and antibody purification can be conducted to obtain an improved yield of the antibody.

Given the fact that the antibody from #6-5 displayed a greater increased ELISA titer than did those of #6-9, we additionally purified #6-5 using PA affinity chromatography (Fig. 4C) and eventually confirmed the antigen-binding activity of the antibody with various concentrations of hMMP9. As a result, the titer increased in an antigen-concentration-dependent manner and its limit of detection (LOD) value was determined as 28.88 ng (Fig. 4D). We compared the LOD value of #6-5 with that of a commercial anti-hMMP9 monoclonal antibody that was developed by a hybridoma cell culture (10327-MM06, Sino Biological), whose LOD value was determined as 293.9 ng. This resulted in a finding of 10-fold higher sensitivity of the antibody produced in this study than the commercial antibody (Fig. 4D).

It should be noted that, contrary to the commercial anti-hMMP9 antibody from hybridoma cells, the antibody developed in this study was produced from CHO cells. Accordingly, repeated immunizations were not necessary for acquiring a new batch of hybridoma cells, which are easily contaminated, and thus have a risk in case of continues subculture. Moreover, as this process employed herein was founded on a stable CHO cell culture, repeated transfection steps were not required, which are necessary for a transient expression system with low label of labor intensity and low cost.

According to the merits of CHO cell-based antibody generation system, including stable producing of antibody with high yield, we herein established a novel CHO cell line for producing recombinant antibody against hMMP and showed its antigen-binding efficiency. This system about CHO cell-based production of a recombinant human MMP9 antibody is potentially important as a therapeutic product for disorders. One week subculture of the cell line established in this study (Additional file 1: Fig. S5), followed by collecting the culture medium and purification, was sufficient to acquire a highly purified antibody (> 99% purity) with the yield of 1.58 mg from 1 L culture. However, the commercial antibody is necessary to be produced *via* hybridoma technology by fusing B cells from hMMP9-immunized mice with myeloma cells. Namely, the simple and rapid CHO cell-based mammalian antibody expression system, which is developed in this study is highly effective for reproducible production of anti-hMMP9 antibody. In addition, the antibody had endotoxin level of 0.71 EU/mL, which was as low as acceptable for preclinical research [20].

## Conclusion

In this study, we developed a recombinant anti-hMMP9 monoclonal antibody that revealed high antigen-binding activity *via* a CHO cell-based mammalian expression system. We confirm the transfection efficiency of antibody-expressing DNA into cells in a convenient manner by using the GFP-expressing gene as a control. However, a flow cytometry-based approach by detecting and/or separating full-sized antibody-expressing cells can be used to evaluate the transfection efficiency further exactly. As CHO cell-based antibody expression is recognized for its high stability, the process defined herein is highly effective for reproducible antibody production. The entire procedure delineated herein can be a beneficial method for acquiring monoclonal antibody-producing CHO cell lines against antigens of interest. After screening a single clone, two weeks were required to obtain a stable expression. We collected the antibodies after passaging the cells 3–5 times and cells were sub-cultivated every 2–3 days with a 1:4 split, indicating the cells were rapidly divided with a generation time of 12–14 h. Furthermore, the information regarding antibody production described herein can be used for developing new direction in innovation about the industrial recombinant antibody expression platform, including host cell line stability for stable long-term production, expression optimization, and effective clone selection system. We expect the use of this antibody in the broad range of therapeutic applications as treatment or detecting agents for inflammatory and cancer-related disease after evaluating further pre-clinical studies, including *in cellular* and *in vivo* research.

## Methods

### Gene cloning

To produce CHO cell-based recombinant anti-hMMP9 antibody, HC and LC genes of the antibody (PDB: 5th9) was inserted into pcDNA3.1(−), respectively (Table 1 and Additional file 1: Fig. S1)). To produce H chain of antibody, a signal peptide gene (caccatgggatggagctgtatcatcctcttcttggtagcaacagctacaggtgtacactcc) followed by HC gene of anti-hMMP9 antibody was synthesized and digested with *NotI* and *HindIII*. Similarly, to produce L chain of antibody, a signal peptide gene followed by LC gene of anti-hMMP9 antibody was synthesized and digested with *NotI* and *HindIII*. Each digested insert gene was ligated to *NotI*- and *HindIII*- digested pcDNA3.1(−), respectively, resulting in pcDNA3.1(−)::anti-hMMP9 HC and pcDNA3.1(−)::anti-hMMP9 LC, respectively.

### Cell culture

Cell culture was performed according to the CHO cell culture method published by Freitag’s group [21] Chinese hamster ovary cells (cell line CHO-K1 (CCL-61, ATCC)) were cultured in an atmosphere of 5.0 % CO_2_ at 37 °C (incubator: Forma SteriCult or Forma Direct-Heat, ThermoFisher Scientific, Dreieich, Germany) with RMPI 1640 medium supplemented with 2 mM L-glutamine, 0.1 mg/mL penicillin/streptomycin and 10 % fetal calf serum. To adapt the adherent cells to the cultivation in suspension, 1 x 10^5^ cellswere plated into a 6-well plate with serum- and protein-free ProCHO-AT medium (Lonza, Verviers, Belgium). After two days, the medium was exchanged to ProCHO4 (Lonza, Verviers, Belgium), which is a medium for inducing adaptation to suspension in CHO cells. Afterwards, the cells were incubated for another 2–5 days. Cells were recovered by centrifugation (200 × g, 5 min) and resuspended in the serum-free production medium ProCHO5 (Lonza, Verviers, Belgium). 1 × 10^5^ cells/mL of cells were inoculated in a spinner flask with 100 mL of ProCHO5 medium. Spinners were placed in the incubator (37 °C, 5.0 % CO_2_) and stirred at 50 rpm.

### Transfection, selection, and single-cell cloning

pcDNA3.1(−)::anti-hMMP9 HC and pcDNA3.1(−)::anti-hMMP9 LC were co-transfected to CHO cell using Lipofectamine3000 (ThermoFisher Scientific, Dreieich, Germany) according to the manufacturer’s protocol. At the same time, we established EGFP expressing control CHO cell line according to the method conducted by Freitag’s group [21]. CHO cells were cultured in a 6-well plate up to the confluency reached 80–85%. and were transfected with each vector. After 48 h, the cells were collected by trypsin treatment and the transfection efficiency was estimated by imaging the EGFP fluorescence using fluorescence microscope (Olympus, Tokyo, Japan). To select the transfected clones, the transiently transfected cells were grown in the medium supplemented with 600 μg/mL antibiotic G-418. To screen for high producing clones, the transfected single cell was transferred into 96-well plates with 100 μL of G-418 containing medium (procedure of limited dilution [6]). When the number of cells in the well is not one, the cells were excluded for the subsequent measurements. Following dilution, the cells were incubated for 2–3 weeks. Twenty plates per transfection were screened.

### Purification of antibody

After screening, positive subclones were grown to larger volumes, and antibodies obtained from each cell supernatant were purified according to our previous experimental method regarding antibody purification [22]. 10 μL of protein A agarose resin was mixed to the 1 mL of cell supernatant. The mixture was incubated at room temperature for 1 h. andwashed three times using 500 μL of PBS. Afterward, 100 μL of 0.1 M glycine (pH 2.5) was added to the sample to elute the antibody from the beads and 10 μL of PBS was immediately added to the eluted sample. The absorbance at 280 nm of the sample was measured using nanodrop and the antibody concentration was calculated.

### Confirmation of the antigen-binding efficiency

Sandwich ELISA for conforming the activity of purified antibody was performed as follows: 200 ng of purified anti-hMMP9 antibody or commercially available mouse anti-hMMP9 antibody (Sino Bio-logical Inc, Beijing, China) was immobilized on the 96-well plate for 16 h at 4 °C, and the well was filled with 200 μL of PBST (PBS buffer with 0.05% Tween 20) that contains 2% BSA for 1 h at 37 °C and washed three times with 200 μL of TBST (TBS buffer with 0.05% Tween 20). Subsequently, 2 ng of hMMP9 (Sino Biological Inc, Beijing, China) was added and incubated for 1 h at 25 °C. After washing three times with 200 μL of TBST, bound protein was probed with 100 ng HRP-conjugated rabbit anti-hMMP9 antibody (Sino Biological Inc, Beijing, China) in TBST for 1 h at 25 °C. The well was washed three times with 200 μL of TBST and developed with 100 μL of tetramethylbenzide (TMB) solution. After incubation for 15 min, the reaction was stopped with 25 μL of 1N H2SO4, and the absorbance was read at 450 nm using a microplate reader Model 680 (Bio-Rad). The dose–response curves were fitted to a four-parameter equation, using GraphPad Prism software.

## Supplementary Information

**Additional file 1.** **Fig. S1.** Schematic representation of the antibody expression vector including the heavy or light chain of anti-hMMP9 antibody. **Fig. S2.** SDS-PAGE analysis of colonies (#3, #6, #10, #13, #14, and #18) after 1st selection of antibody with (left) or without (right) protein reducing. **Fig. S3.** The elution diagram of the size exclusion chromatography of #6-5 and #6-9 of antibodies. **Fig. S4.** The elution diagram of the PA affinity chromatography of #6-5, which was initially purified using size exclusion chromatography. **Fig. S5.** Growth curve of the antibody producing cell line #6-5. **Fig. S6.** Complete original electrophoresis gels.

## Data Availability

All data generated or analyzed during this study are included in this published article.
