# Supplementary material for: Development of a CHO cell line for stable production of recombinant antibodies against human MMP9
Source: BMC Biotechnol. 2022 Mar 7;22:8. doi: 10.1186/s12896-022-00738-6 (PMC8903741; doi:10.1186/s12896-022-00738-6)
Supplement: Supplementary file 1 — Additional file 1. Fig. S1. Schematic representation of the antibody expression vector including the heavy or light chain of anti-hMMP9 antibody. Fig. S2. SDS-PAGE analysis of colonies (#3, #6, #10, #13, #14, and #18) after 1st selection of antibody with (left) or without (right) protein reducing. Fig. S3. The elution diagram of the size exclusion chromatography of #6-5 and #6-9 of antibodies. Fig. S4. The elution diagram of the PA affinity chromatography of #6-5, which was initially purified using size exclusion chromatography. Fig. S5. Growth curve of the antibody producing cell line #6-5. Fig. S6. Complete original electrophoresis gels. [file 12896_2022_738_MOESM1_ESM.docx]

**Supplementary Materials:**


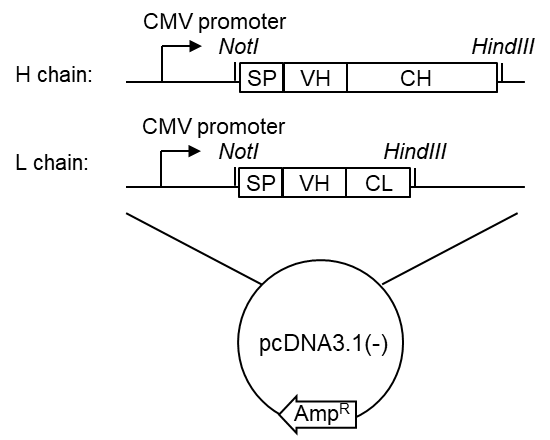


Fig. S1. Schematic representation of the antibody expression vector including the heavy or light chain of anti-hMMP9 antibody.

SP indicates signal peptide.

Fig. S2. SDS-PAGE analysis of colonies (#3, #6, #10, #13, #14, and #18) after 1st selection of antibody with (left) or


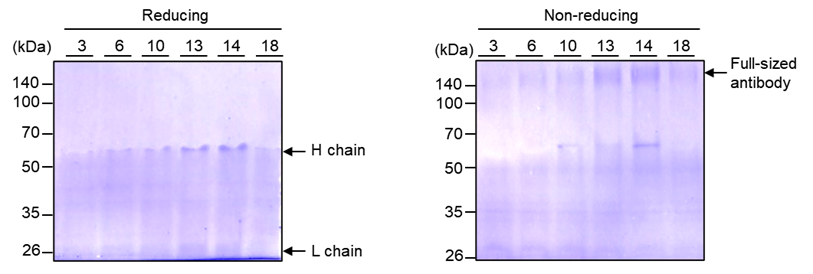


without (right) protein reducing.

Fig. S3. The elution diagram of the size exclusion chromatography of #6-5 and #6-9 of antibodies. Red arrow indicates the peak of expected protein.


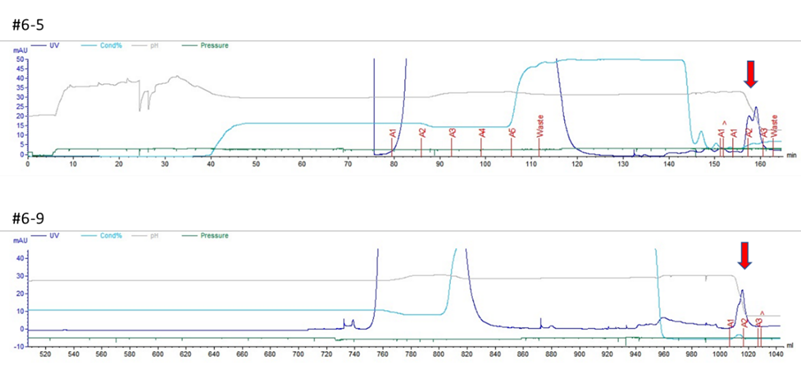

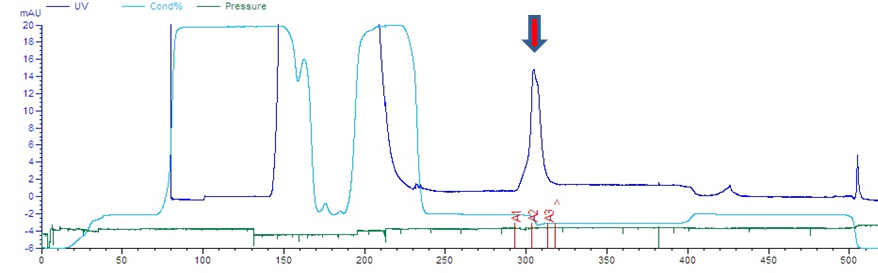


Fig. S4. The elution diagram of the PA affinity chromatography of #6-5, which was initially purified using size exclusion chromatography. Red arrow indicates the peak of expected protein.


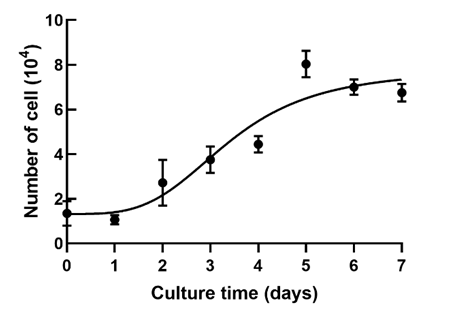


Fig. S5. Growth curve of the antibody producing cell line #6-5. Error bars represent ±1 standard deviation (SD) (n = 4).


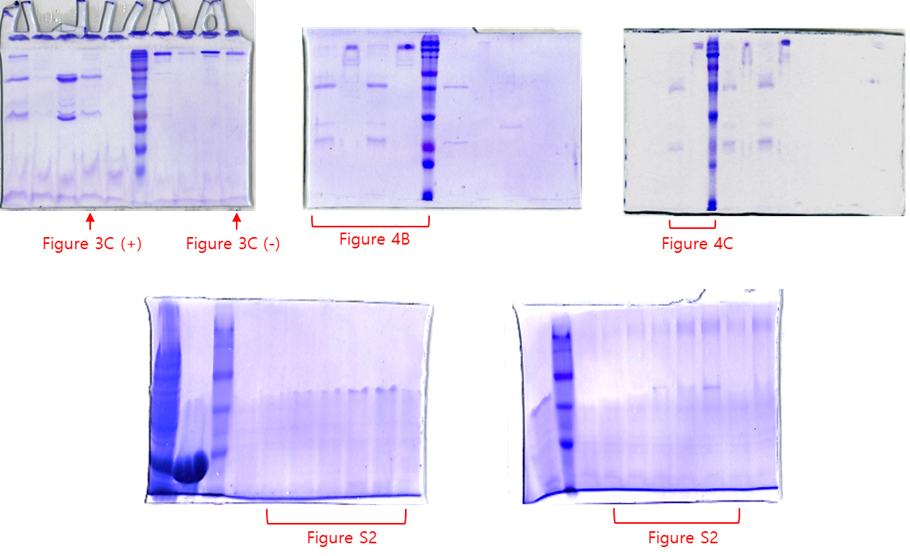


Fig. S6. Complete original electrophoresis gels.
